# The chemical-in-plug bacterial chemotaxis assay is prone to false positive responses

**DOI:** 10.1186/1756-0500-3-77

**Published:** 2010-03-16

**Authors:** Jun Li, Alvin C Go, Mandy J Ward, Karen M Ottemann

**Affiliations:** 1 Department of Microbiology and Environmental Toxicology, UC Santa Cruz, Santa Cruz, 95064, USA; 2 Department of Geography and Environmental Engineering, The Johns Hopkins University, 3400 N Charles St, Baltimore, MD 21218, USA; 3 Department of Earth Sciences, 3651 Trousdale Pkwy, Los Angeles, CA 90089, USA

## Abstract

**Background:**

Chemical-in-plug assays are commonly used to study bacterial chemotaxis, sometimes in the absence of stringent controls.

**Results:**

We report that non-chemotactic and non-motile mutants in two distinct bacterial species (*Shewanella oneidensis *and *Helicobacter pylori*) show apparent zones of accumulation or clearing around test plugs containing potential attractants or repellents, respectively.

**Conclusions:**

Our results suggest that the chemical-in-plug assay should be used with caution, that non-motile or non-chemotactic mutants should be employed as controls, and that results should be confirmed with other types of assays.

## Background

Numerous spatial assays, described below, are used to monitor bacterial chemotaxis to particular chemicals. In these assays, motile bacteria are first placed in a solution that does not contain the test chemical to be analyzed. Next, a high concentration of the test chemical is placed adjacent to the bacterial solution, and allowed to diffuse into it. If the bacteria respond chemotactically, they form either zones of concentrated bacteria or regions of clearing. One widely-used chemotaxis assay is the quantitative capillary assay in which the test chemical is placed in a narrow-bore capillary, and the bacteria are in a surrounding solution [1]. In this assay, chemotactic responses are measured by determining the number of bacteria that move into the capillary. This assay works well for attractants, but not well for repellents as noted by Tso and Adler [2]. These authors first proposed the chemical-in-plug assay, which is now often called the plug-in-pond assay, as a way of studying the effect of repellent stimuli on bacterial behavior. This quick and simple assay places a potential chemoeffector in an agar plug and surrounds the plug with a turbid suspension of bacteria in soft agar. Because the bacteria can swim in the soft agar, a zone of clearing quickly appears around the hard agar plug if the chemical within the plug is a repellent. In their assays, Tso and Adler observed a ring of motile bacteria surrounding the outer edge of the cleared zone and showed that the distance this ring had moved away from the plug was dependent on the concentration of the repellent. A variation on the assay, which places potential attractant chemicals within the hard agar plug, has also been used extensively, again because the assay is technically simple and results can be obtained rapidly. The original study by Tso and Adler used *Escherichia coli *as the model organism, and the assay has since been used to study behavioral responses in an extensive range of bacteria including *Bdellovibrio bacteriovorus *[3], *Campylobacter jejuni *[4,5], *Shewanella oneidensis *[6], *Halobacterium salinarum *[7], *Geobacter metallireducens *[8], and *Flavimonas oryzihabitans *[9]. In the course of our own studies using two completely unrelated bacteria, *Helicobacter pylori *and *Shewanella oneidensis*, we found that non-motile and non-chemotactic mutants form what appear to be chemotactic responses. The usual negative control for these experiments is to note accumulation around a plug lacking any chemical. Here we present evidence that non-motile or non-chemotactic mutants must be used as additional controls to prevent inaccurate conclusions. Several of the above cited studies employed multiple chemotaxis assays, ensuring that conclusions were not based on the results of a single assay. We thus are not calling into question the results of previous studies, but are simply pointing out that misinterpretation of results can be prevented by the judicial use of non-motile or non-chemotactic mutants as negative controls.

## Methods

The *H. pylori *chemical-in-plug assays were done with wild-type *H. pylori *G27 [10] and SS1 [11] and their isogenic mutants. These strains were grown as described [12] in Brucella broth plus 10% fetal bovine serum (FBS), a medium called BB10. After growth, the bacteria were collected by low-speed centrifugation, washed and resuspended in a solution of phosphate-buffered saline with 1% dialyzed FBS (PBS1) and warm 0.3% Bacto agar. The final bacterial concentration was ~6 × 10^7 ^bacteria/ml. This bacterial solution was poured around hard agar plugs composed of PBS1, 2% Bacto agar, and the compound to test. After solidifying, the plates were incubated at 37°C in 10% CO_2_/5% O_2_/85% N_2_. Plates were monitored every 30 minutes for up to four hours. Plates were then placed at 4°C for up to 24 hours until images were captured using a digital camera. There was no change in the appearance of the plates during the 4°C incubation. The doubling time of *H. pylori *is 6-12 hours, so growth inhibitors were not used for these assays.

For *S. oneidensis *chemical-in-plug assays, strains were grown aerobically overnight in Luria-Bertani (LB) medium at 30°C, with shaking. Attractants (20 mM final concentration) were added to molten 1.5% agarose prior to the agarose being poured into Petri dishes. The agarose plates were transferred to an anaerobic chamber (atmosphere 5% H_2_, 95% N_2_) to equilibrate overnight. Plugs were then cut from this agarose for the assay. Two methods were used to prepare the *S. oneidensis *cells, although in both cases approximately 1 × 10^10 ^cells/ml were used in the assays. In the first method, the overnight LB-grown cultures of *S. oneidensis *were mixed at a 1:1 ratio with 1% molten Bacto agar at 50°C, chloramphenicol added to a final concentration of 3.4 μg/ml (to act as a growth inhibitor), and the soft agar containing the cells poured around the equilibrated plugs. In the second method, cells grown overnight in LB were washed with 100 mM HEPES buffer (pH 7.4) to ensure that residual LB medium would not interfere with the assay, then resuspended in fresh 100 mM HEPES (pH 7.4) containing chloramphenicol (as above). The washed and resuspended cells were mixed with 1% molten agar and poured around the hard agarose plugs. The redox indicator dye resazurin was added to duplicate sets of plates to ensure that no oxygen contamination was present prior to the initiation of the assays. Responses were observed and documented after 4 hours. Swim plate assays were performed as outlined in [13].

## Results and Discussion

**Figure 1:**
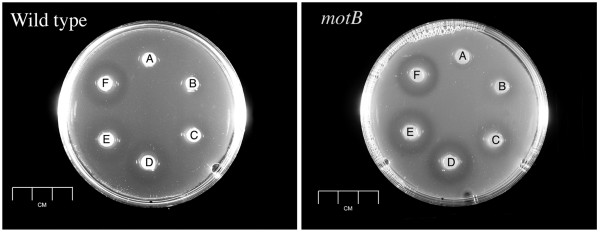
**Chemical-in-plug assays with *H. pylori *wild type G27 (left) and SS1 Δ*motB***. The topmost "A" plug contains no addition (negative control). Proceeding clockwise the plugs contain 1 mM alanine (B), 10 mM alanine (C), 50 mM alanine (D), 100 mM alanine (E), and a 1:4 dilution of *Brucella *broth (F). The assay was repeated six independent times; the plates above show one representative assay. Similar results were obtained with wild-type SS1 and non-chemotactic mutants of both SS1 and G27 (data not shown).

*Helicobacter pylori *is a human gastric pathogen that requires motility and chemotaxis for infection [12,14,15]. There is a limited understanding of what this bacterium uses chemotaxis for; to date, few specific chemicals and properties such as pH or energy status have been determined [16,17]. We thus tried the chemical-in-plug assay to analyze a putative attractant, alanine, which is also required for *H. pylori *growth [18]. After four hours, zones of clearing were apparent around alanine-containing plugs as well as plugs with diluted growth media (Figure 1). *H. pylori *exhibits chemotaxis in soft-agar plates containing the same growth media [for example, [19]]. No clearing was seen around the negative control plugs that contained only PBS1+Bacto Agar. The clearing response could first be seen after two hours, and the clearing zone increased throughout the duration of the assay. Additionally, the degree of the response was concentration dependent, with larger clearing zones at high alanine concentrations (Figure 1). We saw similar responses with two *H. pylori *strains, SS1 and G27 (data not shown), and thus proposed initially that this bacterium responds chemotactically to alanine. The zone of clearing observed around plugs containing alanine, or *Brucella *broth, is reminiscent of a repellent response, as the *H. pylori *leave the vicinity of the alanine, although no ring of cells was observed at the edge of the clearing zone.

Both chemotaxis and motility are required to form chemotactic responses, so we tested *H. pylori *mutants defective for these processes in our assay. The non-motile *H. pylori *mutant used has the *motB *gene deleted. The MotB protein is a motor protein required for flagellar rotation; mutants lacking it are flagellated but the flagella cannot turn. This mutant has been well characterized [12] and does not recover motility. This mutant, surprisingly, formed the same clearing zones as wild-type *H. pylori *(Figure 1). To further substantiate this finding, we repeated the assay with multiple characterized non-chemotactic and non-motile mutants generated in two strain backgrounds, G27 and SS1, including *cheW*, *cheA *and *cheY *mutants [20], and found consistent zones of clearing in all strains tested (data not shown). These findings suggest that the zones of clearing are independent of chemotaxis and motility.

These findings with *H. pylori *prompted us to examine whether other bacteria might similarly display false-positive responses in the chemical-in-plug assay. Previously, chemical-in-plug and swim plate assays had been done using *S. oneidensis *strain MR-1, with the finding that this microbe responds chemotactically to a number of anaerobic electron acceptors [6,21,22]. Subsequently, the genomic sequence of *S. oneidensis *strain MR-1 allowed us to construct and characterize an isogenic non-chemotactic mutant lacking the chemotaxis kinase CheA (Δ*cheA-3*) [13]. This mutant retains wild-type swimming speed, but is unable to reverse direction. The mutant also does not show chemotaxis to anaerobic electron acceptors in either the swim plate or capillary assays [13]. However, no report on the behavior of this mutant in the chemical-in-plug assays was previously made.

**Figure 2:**
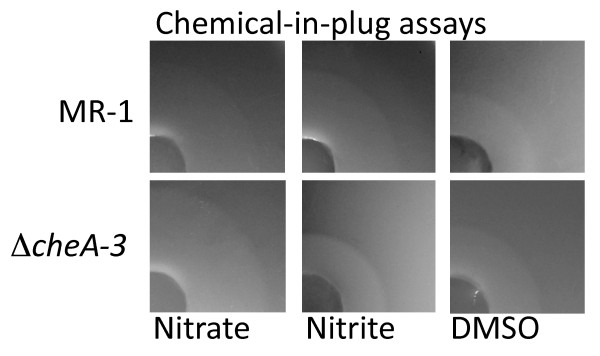
**Chemical-in-plug assays with *S. oneidensis***. The notation on the left indicates the strain used, and each column represents a different chemical in the plug, as indicated below each set of panels. The plugs for the assays are visible in the lower left hand corner of each photograph.

In this study, we report that both *S. oneidensis *MR-1 and the Δ*cheA*-3 mutant show zones of accumulation around all of the plugs containing anaerobic electron acceptors, but not around the control plugs, in the chemical-in-plug assay. Specifically, nitrate, nitrite, and DMSO (Figure 2), plus TMAO and fumarate (not shown) elicited responses from both wild type and the Δ*cheA*-3 mutant. Responses were stronger for the unwashed cells (not shown), although the cells washed and resuspended in 100 mM HEPES still displayed zones of apparent cell accumulation (Figure 2), although in neither were rings of cells apparent at the edge of the accumulation zones. We confirmed that the Δ*cheA*-3 mutant had not reverted to wild-type by performing swim plate assays. As shown previously, the Δ*cheA*-3 mutant was unable to respond to electron acceptors in this assay [13]. Because cells that are unable to reverse their direction of swimming would be expected to be non-chemotactic, the results of the swim plate and capillary assays performed previously with the Δ*cheA*-3 mutant [13] are more likely to be correct than the results of the chemical-in-plug assays. Consequently, it appears likely that the chemical-in-plug assay generates false positive results when used for analyzing *S. oneidensis *motility behavior.

## Conclusions

In summary, our analyses show that both non-motile and non-chemotactic mutants can display what appears to be chemotaxis in the chemical-in-plug assay. We observed these motility-independent responses in two unrelated microbes, *H. pylori *and *S. oneidensis*, suggesting it is a common phenomenon in response to several compounds. While both microbes displayed motility-independent responses, the characteristics of each were different. *S. oneidensis *had what appeared to be bacterial accumulation around the plugs. A close observation of these zones, however, found them to be white in color rather than the more typical pinkish color of this bacterium. Consequently, it seems possible that the bacteria respond to the chemical gradients emanating from the plugs by forming a precipitate that can be confused with a behavioral response. The recent study by Baraquet *et al. *[23] suggests that the formation of this precipitate, if that is what it is, is dependent on respiration because mutants unable to respire anaerobically did not show accumulation when anaerobic electron acceptors were used as the attractants. The *H. pylori *response, in contrast, consists of actual clearing around the plugs. It is possible that the clear zones arise from bacterial lysis, although this seems unlikely given the rather mild nature of some of the plug contents (e.g. 1/4 Brucella broth). The lack of rings of cells at the edges of the zones of clearing and accumulation may be an indicator of a false chemotactic response. However, we did not explore the basis for this response further.

Choosing appropriate chemotaxis assays for studies involving different microorganisms is complex. For example, *H. pylori *has displayed chemotaxis in a liquid-based microscopic assay [16], in commercial chemotaxis chambers [17] and in the agarose-in-plug bridge assay [T. M. Andermann and K. M. Ottemann, unpublished and [24]]. Some scientists have also reported success with *H. pylori *capillary assays [25,26], but our own experience has found this assay to be unreliable (unpublished). For *S. oneidensis*, the swim plate and capillary assays seem reliable, as does a microscopic version of the chemical-in-plug assay where bands of motile cells in liquid have been filmed moving towards or away from plugs containing chemoeffectors (unpublished). However, our conclusion is that the chemical-in-plug assay should only be employed, in combination with other assays, if suitable non-chemotactic or non-motile mutants are available to act as negative controls.

## Competing interests

The authors declare that they have no competing interests.

## Authors' contributions

JI, ACG, MJW and KMO designed the experiments and interpreted the data. JI and ACG performed the experiments. MJW and KMO wrote the manuscript. All authors read and approved the final manuscript.
